# How to get most out of a Medical Conference, and Love it too!

**DOI:** 10.12669/pjms.36.4.2628

**Published:** 2020

**Authors:** Chaudhry Aqeel Safdar

Most of us have attended medical conferences and will continue to do so. I love attending them. Everyone has his own motives and ideas about attending these events.^1^ Many come back with nothing more to show than a conference bag. I come back, refreshed, motivated, wiser and with plenty of new ideas and friends. On returning I usually cannot wait to discuss with my colleagues and trainees and share the ideas and wisdom that I gleaned from the sessions, fringe meetings or over a cup of coffee during breaks.

A conference can be the chance of a lifetime to meet some of the big names, even world leaders in your chosen field or medical science in general. Medicine has its fair share of pompous prigs, but there are many brilliant scientists who are human and friendly. With a few carefully placed queries, you may show your shared interests, and this can grow into a friendship or even collaboration.^2^ It is important that before you think about going to a conference you should have a plan. Be well prepared, ready to learn, network and have fun.

## Which Conference?

In selecting a conference, a number of factors are important. Do you always attend conferences related to your field? These are most useful for learning and sharing new ideas; how people are tackling old problems, learning from the masters; keeping you involved in the proceedings and perhaps picking up a trick or two about your day to day clinical irritants. But once in a while you may want to attend a meeting which is allied to your specialty or even completely alien. I am a paediatric surgeon, but I try to attend meetings held by paediatricians, general surgeons and medical educationalists. It enhances your circle of colleagues, projects your specialty and your department, but most of all generates ideas from a different perspective. Sometimes it is also fun not to know anyone at the meeting, and then you can be a true “conference tourist”; own time, own pace, own place.

## National or International?

This is an important decision. National has the advantage of meeting and catching up with friends and colleagues, most of whom you would know. Issues discussed are relevant and suggestions do-able; easy to manage logistically, financially and socially. But if you can afford it, manage the visas, can stay away from work (and family!) for a rather longer period, international congresses can be invigorating (Spoiler Alert: Check the Covid Situation). No day to day putting out of fires in hometown, detached, able to completely chill and all the while meeting “world-authorities”. You may be the first one in listening to ideas which will make into the textbooks quite a few years down the line. You may be able to explore a new culture or country. Taking your family along is not a bad idea. They deserve a break too, and you can spend that elusive quality time with them. But a word of caution: try to pay for this from your own pocket; you don’t want any strings attached, fingers pointed and without a guilty conscience. After all, isn’t that’s why you are working so hard and earning?

## Why do you want to attend?

Have clear objectives: *Education*- to update knowledge; *Inspiration*- to hear new ideas and meet professional leaders; *Evaluation*- to review your own work and plan for the future; *Presentation*- a platform for your own research; *Recreation*- to relax and enjoy the company and culture around with your loved ones!^2^ The interesting fact is that all these objectives are not mutually exclusive. You can achieve all of these. Prioritize and Equalize!

Distance lends enchantment or a perspective to your work. When we are caught up in the day to day grind at home, you have no time for an honest reflection. Innovations, perhaps as simple as a cork, may be apparent only when you are not frantically bailing a leaky boat.^2^ A good conference has the capacity to bring you, no matter your career stage, out of a slump.^3^ Also make an effort to join the hosts at cultural events, most of whom welcome spouses. I am sure a short post-conference holiday can also be appended.

## Do you have a Plan?

I always register in advance. Don’t think about trying to sneak in without paying. Or wait to the last day, thinking that it may come from ‘somewhere’. At national conferences, just turning up and avoiding the usually small payment, doesn’t look very nice. The organizers may not say anything, but they do talk. Don’t throw your weight around. This doesn’t work at international conferences; so if you have decided utilize the early bird offer; this saves money and gives you time to work out logistics. These are usually quite expensive, and if your organization is willing to support, as in other countries, apply for it. But register in advance. Apply for the visas early and complete formalities; ask for the invitation letter from conference secretariat, if required.

If you have something worthwhile to present, don’t hesitate. Most international moots would like an international representation from varied countries and societies. They may surprise you by accepting. It is the most prestigious achievement for a professional to represent his country or organization. Of course, then get one prepared and rehearsed the best you can.

Now whether it is a conference in your backyard or on the other side of the world, you must have a good plan; research well in advance. You must be ready to get the most out of this precious break. Find out what the conference will include; reflect on your intellectual needs and pre-register for any interesting workshops. Find out about the hosts, the speakers and the attendees. When you go in with this information you will be ready to get the most out of the conference.

## Next, what to pack?

You need to know what the expectations for wardrobe will be. Will there be formal events? Do you need to wear a specific type of clothing for the educational or social events? Make sure you know and plan your packing accordingly. Check the weather of the venue, for that time of the year; it may be entirely different from your home weather.^4^ Carry an extra charger, a power-bank (can get you lot of cheers from someone whose mobile ran out of juice at a crucial moment), spare flash-drives, a power plug adapter (the type easily checked on internet). If travelling abroad, do you need to take some special documents? Your travel agent will know. Do take your own supply of paracetamol and your personal concoctions for coughs, colds and upset tummies; and don’t forget spare glasses. Have you told your credit card vendors about using them abroad? Roaming on your mobile is a good idea; it helps till you get a local SIM. Download Tripadvisor and Google Maps onto your mobile.

## Where to stay?

Best place to stay is at or very near the venue. Its benefits far outweigh the extra expense, if not too exorbitant. You can nip up to your room for a snooze, sleep till the last moment in the mornings, meet and greet other participants at buffet breakfast and keep the family involved, all the while. If not practical, then look for a place at a walking distance. The money you save on taxis and travel will far outstrip the hotel rate.

Next, revise and revisit your ***conference goals***; Take a good supply of your business cards, with your personal number and email. For my junior colleagues, don’t be afraid to take the lead and introduce yourself to those around you. When it’s a social or academic event, this is expected and you should lead with a smile and a strong handshake.^3^

## At the Conference

Arrive a day earlier and get a good night’s sleep. Iron out any settling down wrinkles. Check the time for arriving at the venue and plan how to get there; allow extra time on the first day. Benefits of staying at or close to the venue would be obvious and you can thank me for that later. Hopefully, you would have already received or downloaded the programme, and made an outline of what to attend and where. Have a look at the venue layout if available and familiarize. Some conference books are a puzzle of maps, abstracts, advertisements, codes, numbers, and cross references that make a Mensa test look simple.^2^

If you are a presenter check the instructions for submitting the slides. Always keep a copy on a USB and send a copy to yourself by email or on Cloud, which can be retrieved if nothing else works at the last moment. Always have your own laser pointer and slide-presenter; theirs may stop working at the last moment. It’s fundamental to familiarize yourself with the technical paraphernalia.

## What to wear?

Depending on your status and age, dress properly, and according to the norms of the host country and society. Closed collars, suit or appropriate combination. Come across as a professional. Ensure the clothes are comfortable. You don’t want to be thinking about those hurting toes in new shoes while trying to concentrate on the talk.

There is often a long queue at the ***registration*** desk. Be polite and supportive to the organizers; they are already under a lot of pressure. Don’t be grumpy! After registering and obtaining the name tags, grab the latest programme and abstract book. Leave the conference bag to be collected later in the day. It will save you the hassle of carrying it and looking after it all day.

If you have identified beforehand where to go first, just proceed. Be on time. The greetings and huggings can wait or continue side by side. ***Where to sit?*** I prefer to sit a couple of rows from the front, ensuring a good view of the presenter and the screen. If you have to leave early to attend another session try to sit near the aisle. Have the courtesy to leave when one presentation is concluded. The mobile would be on silent or turned off and avoid the temptation to look at the messages and missed calls every two minutes. I use the memo feature on my mobile to take any notes and add a picture of the title and presenter, if allowed. The abstract book is your core source for post-conference review and debrief. So guard it carefully. I write encrypted notes in the margins opposite the presenter’s abstract for later recall. If there are simultaneous sessions, and have something not to be missed, recruit a colleague or junior and divide the sessions among you. Later on notes can be exchanged. If polite, avoid sitting with someone you know; it will help you concentrate and preempt irritation of continuous whispering; both to you and to those around you.

So what am I listening to and what am I thinking? No doubt you want to listen to the content, and internalize the message. But also pay attention to a good presenter’s style, body language, delivery, slides, appropriate humour, the ‘hooks’ to keep the audience engrossed, the showmanship, the handling of questions and how they exude their charisma. Also learn from the not so good presenters – thinking all the while that this is what I would not be doing! Not facing the audience, awkward hands, forgetting the sequence, inadequate preparation, borrowed material, language, dress – the list goes on and on. All the improvements I have incorporated in my talks have been gleaned from various genres of presenters. Try to meet the speakers, when convenient, to discuss and appreciate their work and any clarifications. Say something nice to young speakers; be honest and give polite feedback for the sake of their improvement. Use your skills of constructive feedback.

Probably the biggest mistake I see people (including a younger version of myself) make at conferences is only hanging out with your close friends and ward-mates. It is more fun, but it means missing out on the opportunity to build new bridges. Never eat lunch exclusively with your colleagues and pals – acquire new ones and learn from their ideas and knowledge. Go easy on what you eat; something easy to digest, not too spicy; avoiding dodgy foods which can save you embarrassing trips to the washrooms; but be brave to try something new and local; it adds to your international culinary wisdom.

Before you ask to have pictures taken with famous participants, consider the cultural norms of the meeting; don’t hound them. Selfies can be good fun, but perhaps could be reserved for your very close circle.

Opening and closing ceremonies may be considered a waste of time, but they help you identify some important people on the stage. The ***commercial exhibition*** is also an important source of the latest. Get the contact numbers of the vendors who interest you. Make time for the ***Cultural events***. They are usually entertaining and the families have an opportunity to meet your new found friends informally and feel involved.

You know yourself best, but don’t underestimate the power of a good night’s sleep, or a snooze. Rarely it’s okay to jump Ship.^5^ Make time for yourself!

## Time to return home?

After you have returned and thanked your stars that your bags have arrived with you and not gone to the *ether* world, don’t lose your conference frame of mind too soon. Give an informal presentation to your department, soon. The sooner the better, while it is still fresh in your mind! This is where your precious programme and abstract book with margins full of notes will be most handy.

Keep in touch with the acquaintances you made at the medical conference using emails, tweets, and LinkedIn messages. If you think this has no benefit, consider this—all it takes is one new relationship to change your career. I believe in serendipity. So send a thank you note, and follow up emails, as soon as possible. You may not be in the pictures with the ‘bride and groom’, but a thank you note is sometimes more effective. All that is left now is to print and save your CME certificates before the conference site deadline. *Now, start planning for the next conference*.
